# Primary epithelioid angiosarcoma of the breast masquerading as carcinoma

**DOI:** 10.3747/co.v17i1.451

**Published:** 2010-02

**Authors:** S. Muzumder, P. Das, M. Kumar, S. Bhasker, C. Sarkar, K. Medhi, V.K. Iyer, G.K. Rath

**Affiliations:** * Department of Radiotherapy, Institute Rotary Cancer Hospital, All India Institute of Medical Sciences, New Delhi, India; † Department of Pathology, All India Institute of Medical Sciences, New Delhi, India; ‡ Department of Medical Oncology, All India Institute of Medical Sciences, New Delhi, India

**Keywords:** Breast, primary epithelioid angiosarcoma

## Abstract

Here we report a case of primary epithelioid angiosarcoma (eas) of the breast occurring in a 30-year-old woman. Following fine-needle asspiration cytology (fnac) and tru-cut biopsy, the patient was initially diagnosed with mammary carcinoma and thereafter underwent modified radical mastectomy. Postoperative histopathologic examination and immunohistochemistry revealed a diagnosis of primary epithelioid angiosarcoma of the breast. The patient received postoperative radiotherapy to the chest wall and was started on adjuvant thalidomide. Preoperatively, eas can be mistaken for carcinoma because it is difficult to appreciate the typical morphology on fnac or tru-cut biopsy. Indeed, this is an area of potential diagnostic error because, nowadays, neoadjuvant therapy is often instituted after core biopsy of a breast mass. This case is being reported not only for its diagnostic difficulty, but also because of its rarity in English literature.

## 1. INTRODUCTION

Sarcomas represent fewer than 1% of primary breast malignancies 1,2. The most commonly reported primary non-phyllodes sarcomas of the breast are angiosarcoma, fibrosarcoma, malignant fibrous histiocytoma, liposarcoma, and leiomyosarcoma 2. Angiosarcomas account for fewer than 0.05% of all malignant mammary tumours 3.

Angiosarcomas of the breast are commonly seen secondary to radiation therapy; they are also associated with postoperative lymphedema (Stewart–Treves syndrome). In comparison, primary angiosarcomas are relatively rarer. Secondary angiosarcomas arise from skin and show a pattern of infiltration into breast from skin and subcutaneous tissues. In contrast, primary angiosarcomas arise from mammary tissues and infiltrate the skin only in advanced cases. The grading of the tumour is thought to affect prognosis more in primary angiosarcomas; it has questionable value in prognosticating secondary angiosarcomas 4.

Primary epithelioid angiosarcoma (eas) is distinctively rare in the breast and was first reported by Weiss and Enzinger in 1982 5. It has been more frequently reported in other sites such as the skin 6, uterus 7, small intestine 8, lung 9, thyroid 10, and central nervous system and orbit 11. About 100 cases of secondary angiosarcoma of the breast after breast-conserving therapy have been reported, but primary eas of the breast is rare 12. After an extensive literature search using key words such as “eas,” “breast,” and “primary” in PubMed, Scopus, Ovid, and IngentaConnect, we could identify only 9 cases of primary eas of the breast 13–16.

The striking preoperative difficulty in diagnosing these lesions may be attributable to a limitation in cells or tissue yielded by fine-needle aspiration cytology (fnac) and tru-cut biopsy. A misdiagnosis of carcinoma may lead to delay in initiating the aggressive management needed, given that angiosarcomas are the most malignant of all breast tumours 17.

We were recently confronted with a case of nonmetastatic primary eas of the breast that was diagnosed preoperatively as infiltrating ductal carcinoma. Here we discuss the clinical, pathologic, and immunohistochemical features of the case with an intent to convey the diagnostic dilemmas involved.

## 2. CASE DESCRIPTION

A 30-year-old premenopausal woman presented to a peripheral hospital with a 15-day history of a painless lump in the right breast in which fnac was suggestive of carcinoma. This patient was then referred to the breast cancer clinic at our institute. On physical examination, a lump measuring 6×5 cm was detectable centrally in the right breast. This lump was hard in consistency and not fixed to the underlying structure. Overlying skin was unremarkable. No axillary or supraclavicular lymph nodes were palpable. The contralateral breast and axilla were normal.

As initial screening, a fnac was advised, followed by tru-cut biopsy of the lump. A diagnosis of high-grade carcinoma was suggested in both tests. Immunohistochemical staining for cytokeratin was positive in tumour cells; staining for vimentin was negative. The cells were negative for the estrogen and progesterone receptor immunostains, but 2+ positivity for the human epidermal growth factor receptor [her2/*neu* (ErbB2)] was noted. Chest radiography, abdominal ultrasonography, and bone scan did not reveal any metastatic disease.

Based on the overall preoperative workup, a diagnosis of carcinoma of the right breast T3N0M0 was made. A preoperative mammogram was not done, because right modified radical mastectomy (mrm) followed by adjuvant therapy was planned for the patient and performed in July 2008. Intraoperatively, the lump was close to deep fascia; a cuff of pectoralis major muscle was therefore excised along with tumour. The axillary dissection proceeded to the level 3 lymph node, because multiple lymph nodes, the largest being 1.5 cm, were present at levels 1 and 2.

### 2.1 Pathology Findings

#### 2.1.1 FNAC

**FIGURE 1:**
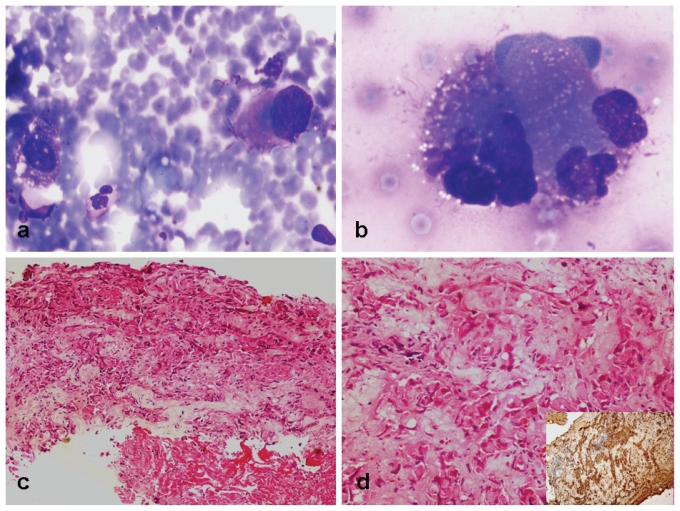
(a,b) Fine-needle aspiration cytology of the breast lesion showed singly-lying epithelioid cells with irregular pleomorphic nuclei and moderate-to-abundant bluish cytoplasm [Giemsa stain: (a) 200×, (b) 400×]. (c,d) Tru-cut biopsy of the breast showed irregular cords and singly-lying pleomorphic cells with moderate cytoplasm and focal necrosis [hematoxylin and eosin stain: (c) 40×, (d) 100×]. (d, inset) The cells were immunopositive for cytokeratin (40×).

On aspiration of the lesion, cellular yield was moderate, comprising predominantly single cells with occasional cell clusters. The tumour cells were noted in a background of blood, together with scattered histiocytes and neutrophils. The tumour cells were epithelioid with eccentric nuclei, coarse chromatin. and moderate-to- abundant cytoplasm with an indistinct margin. The nuclear margin was irregular with identifiable nuclear indentation. Fine cytoplasmic vacuolations were identified. Based on morphology, a possibility of high-grade malignancy was considered [Figure 1(a,b)].

#### 2.1.2 Tru-Cut Biopsy

Based on a suggestion from the fnac report, a tru-cut biopsy was planned. The biopsy comprised one core showing high-grade malignancy with epithelioid cell morphology. The cells were raggedly infiltrating the fibrocollagenous stroma. Focal hemorrhage was noted, but no definite identifiable vascular lumen was seen. The cells had moderate eosinophilic cytoplasm. Few mitotic figures and areas of focal necrosis were seen. No definite ductal pattern or residual benign ducts were noted in this biopsy [Figure 1(c,d)]. An immunohistochemical panel including cytokeratin (ck); vimentin; estrogen (er), progesterone (pr), and her2/*neu* receptor proteins; and thyroid transcription factor was applied. Among these, ck and her2/*neu* were positive in tumour cells, and a diagnosis of primary breast carcinoma was given.

#### 2.1.3 MRM Specimen

The gross resected mrm specimen measured 10×11×6 cm. On sectioning, a spongy hemorrhagic tumour measuring 7×6×2.8 cm was seen centrally and in the upper outer quadrant. The tumour was close to the pectoralis fascia.

**FIGURE 2:**
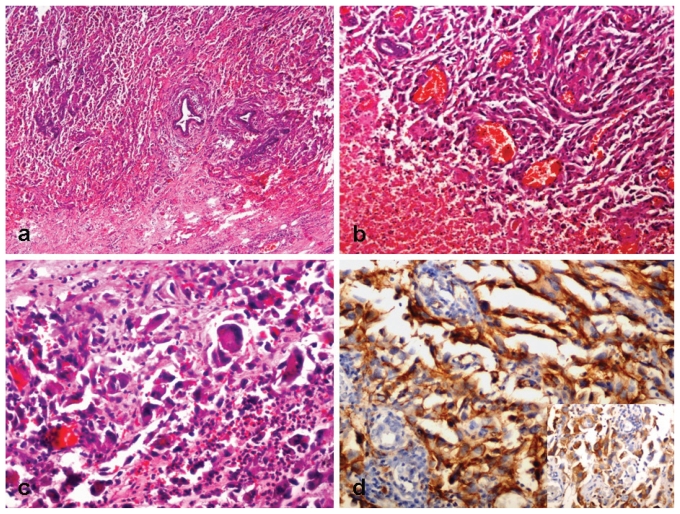
(a) Low-power photomicrograph of the resected tissue shows an infiltrating malignant tumour entrapping the residual ductules (hematoxylin and eosin stain, 40×). (b) The tumour is highly vascular, comprising many large and slit-like vascular spaces (hematoxylin and eosin stain, 200×). (c) The slit-like spaces are lined by pleomorphic epithelioid malignant cells. Few giant cells are noted (hematoxylin and eosin stain, 400×). (d) The tumour cells are strongly immunopositive for CD31 (200×). (d, inset) Immunostaining for the human epidermal growth factor receptor (her2/*neu*) was positive in tumour cells (200×).

Multiple sections showed features of a poorly differentiated malignant tumour with entrapped benign ductules at the tumour periphery [Figure 2(a)]. The tumour was composed of pleomorphic epithelioid cells, multinucleated giant cells, slit-like vascular spaces, fresh and old hemorrhage, necrosis, and scattered inflammatory cells [Figure 2(b)]. The epithelioid cells showed indented hyperchromatic nuclei and a moderate amount of eosinophilic cytoplasm [Figure 2(c)]. Mitotic activity was identified. These cells were seen lining the vascular spaces.

The tumour cells were immunopositive for CD31, and 2+ immunopositivity for her2/*neu* was noted [Figure 2(d)]. The cells were negative for CD34 and the er and pr immunostains.

Overall features of the tumour were compatible with an eas of the breast. The overlying skin, nipple, areola, and deep resected margins were free of tumour. All 13 lymph nodes dissected were free of tumour.

Postoperative computed tomography imaging of the chest (for ruling out lung metastasis) was normal. Left mammogram showed a normal study. The patient was planned for adjuvant radiotherapy and chemotherapy. She received chest wall telecobalt radiotherapy, 50 Gy in 25 fractions over 5 weeks, using two tangential fields. She tolerated radiotherapy well, with grade 1 skin reactions.

Because the role of chemotherapy is not well defined in this disease, the patient was started on thalidomide 100 mg daily based on previous experience at our institute^18^. She developed asymptomatic rashes over her thighs, which resolved spontaneously. She is clinically disease free at 11 months after surgery. This patient has been receiving thalidomide for 10 months and is tolerating it well. We are planning to continue thalidomide till disease recurrence or intolerance to thalidomide.

## 3 DISCUSSION

Epithelioid angiosarcoma is a rare variant of angiosarcoma described in various sites. Only 4 individual cases of primary eas of breast have been described previously 13–16, and 5 cases were mentioned in a clinicopathologic series reported by Nascimento *et al.* 3 of 49 cases of primary angiosarcoma of breast. The tumour is most common during the third and fourth decades of life. It may present as a small painless lump or even as a large hemorrhagic mass. In advanced cases, there may be skin involvement, ulceration, and bleeding. Table I enumerates the clinical, pathologic, and immunohistochemical features and management of the cases that have been described in detail in the English literature (excluding the cases mentioned by Nascimento *et al.*).

Epithelioid angiosarcoma is composed predominantly or exclusively of large, rounded “epithelioid” endothelial cells with abundant amphophilic or eosinophilic cytoplasm and large vesicular nuclei. Initial preoperative incisional biopsy can lead to a misdiagnosis of ductal carcinoma because of similar histopathology. Both diseases can show solid sheets of polygonal cells with intracytoplasmic clear spaces or vacuoles. Only primary eas shows slit-like vascular spaces, which are lined with pleomorphic epithelioid malignant cells. The differential diagnosis of primary eas of the breast includes ductal carcinoma and other poorly differentiated sarcomas^19^.

In our case, the diagnosis was missed on the fnac and tru-cut biopsies, and eas of breast is known to be able to mimic a high-grade carcinoma in a fnac smear or tru-cut biopsy core. Although the cells in our case had nuclear indentation, the absence of cytoplasmic vacuolations, vesicular nuclei, and identifiable vascular channels in the tru-cut biopsy core led us away from the correct diagnosis^20^,^21^

Immunohistochemistry is an important adjunctive procedure in the diagnosis of angiosarcoma—particularly for poorly differentiated forms in which vascular channel formation is difficult to identify. Angiosarcomas express (to a greater or lesser degree) the usual vascular antigens, including von Willebrand factor, CD31, and CD34. Although von Willebrand factor is the most specific of the vascular markers, it is also the least sensitive, often present in a few angiosarcomas as weak focal staining. On the other hand, CD31 combines both relative specificity with excellent sensitivity, and it is positive in approximately 90% of angiosarcomas of all types. Cytokeratin is present in about one third of soft-tissue angiosarcomas, particularly the epithelioid subtype, reflecting the fact that ck cannot be used as an absolute discriminant between angiosarcoma and carcinoma. Epithelioid angiosarcoma is a variant that is positive for CD31, but it is classically negative for CD34, which is another marker of endothelial differentiation. In this case, the cells were negative for vimentin, er, pr, and Bcl2. Many cases express ck along with endothelial markers. The principal significance of those markers is the close resemblance they share with carcinoma. Immunohistochemical overexpression of her2/*neu* in breast carcinomas is described as a predictor of response to alkylating agents or anthracycline therapies; however, its definite role in primary eas of breast remains to be explored 22. In our case, 2+ immunopositivity for her2/*neu* was seen in tumour cells from both the tru-cut biopsy and the postoperative specimen.

An epithelioid morphology can also be found in other vascular tumours that vary considerably in their presentation and behaviour. Low-grade lesions such as epithelioid and spindle-cell hemangioendothelioma, and benign lesions such as epithelioid hemangioma, also appear in the differential diagnosis, as does a pleomorphic carcinoma^21^. Epithelioid angiosarcoma is sometimes difficult to distinguish from epithelioid hemangioendothelioma. However, the presence of a solid growth pattern with necrosis and mitotic activity should generally be regarded as a diagnostic clue in favour of epithelioid angiosarcoma^22^. The distinction from metastatic carcinoma, melanoma, and proximal-type epithelioid sarcoma is based particularly on immunohistochemistry and a relative rarity of such lesions in this primary site. Epithelioid angiosarcoma may mimic the angiomatous variant of epithelioid sarcoma both in morphology and by the occasional expression of ck. However, angiosarcoma is more pleomorphic and usually expresses CD31 together with factor viii. Differentiating the epithelioid variant of angiosarcoma from the usual angiosarcoma depends on typical cell morphology: nuclear indentation and immunonegativity for CD34 stain^22^.

The recommended therapy for primary angiosarcoma of the breast is simple mastectomy, because wide excision alone is associated with high local recurrence rates^23^. Axillary lymph node dissection is not recommended, because the involvement of lymph nodes is extremely rare 23. According to the series reported by Nascimento *et al.,* primary angiosarcoma of breast showed a high rate of metastasis and mortality regardless of tumour grade^3^. In that series, the authors reported no subset analysis with respect to the epithelioid variant of angiosarcoma, of which 5 cases were seen. The most common sites of metastasis are lung, bone, liver, and skin. The roles of adjuvant radiotherapy and chemotherapy are not well defined in primary angiosarcoma of breast.

Primary eas of the breast, a rare variant of angiosarcoma, is a highly aggressive malignancy associated with poor survival. In the 4 reported cases, 1 developed local recurrence, 1 developed axillary recurrence, and the remaining 2 developed distant metastasis. In our case, chest wall radiotherapy was given in view of the large size of the tumour and the intraoperative finding of deep fascia involvement. Adjuvant thalidomide 100 mg daily was started after surgery, because the benefits of other chemotherapy are doubtful, and a case of complete response to thalidomide in angiosarcoma of the breast has been reported from our institute^18^.

## 4 CONCLUSIONS

We report a rare and aggressive case of primary eas of the breast—a disease that can be misdiagnosed as carcinoma because of similar histopathology. An immunohistochemical panel including ck, vimentin, CD31, CD34, er, pr, and her2/*neu* should be used to differentiate lesions with a similar histomorphology to reach a final diagnosis. Mastectomy with adjuvant radiotherapy and chemotherapy appears to be the best treatment modality. Thalidomide appears to be a promising drug in the management of angiosarcoma. Exploration of newer agents is warranted to improve survival.

## Figures and Tables

**TABLE I:** Cases of primary epithelioid angiosarcoma of the breast^a^

Reference	Patient		Treatment	Immunohistochemistry [positive (+), negative (−)]	Outcome
	Age (years)	Sex			
Martinez *et al.,* 1997^13^	26	F	Modified radical mastectomy, postoperative radiotherapy (4860 cGy), doxorubicin and dacarbazine	Vimentin+, factor viii+, CD31+, cytokeratin−	Alive at 7 months, with local recurrence
Farina *et al.,* 2003^14^	49	F	Modified radical mastectomy, no adjuvant	Vimentin+, factor viii+, CD31+, CD34+, cytokeratin−	Died at 15 months of metastasis
Carter *et al.,* 2005^15^	33	F	Simple mastectomy, no adjuvant	nr	Alive at 7 months, with axillary recurrence
Wang *et al.,* 2007^16^	20	M	Complete excision, no adjuvant	Vimentin+, factor viii+, CD31+, CD34+, cytokeratin+	Died at 6 months of metastasis
Muzumder *et al.* (present case)	30	F	Modified radical mastectomy, postoperative radiotherapy (5000 cGy), thalidomide	Vimentin+, CD31+, CD34−, her2/*neu* (erbB-2)+, er−, pr−, cytokeratin−	Alive at 9 months, free of disease; on thalidomide

a Excludes cases mentioned by Nascimento *et al.,* 2008.^3^

F = female; M = male; nr = not reported; her2/*neu* = human epidermal growth factor receptor; er = estrogen receptor; pr = progesterone receptor.
